# Approaches to needle navigation in interstitial brachytherapy using infrared tracking and radiography

**DOI:** 10.1002/acm2.70496

**Published:** 2026-01-31

**Authors:** Veronika Kreß, Ricarda Merten, Christoph Bert, Vratislav Strnad, Rainer Fietkau, Stefanie Corradini, Andre Karius

**Affiliations:** ^1^ Department of Radiation Oncology Universitätsklinikum Erlangen, Friedrich‐Alexander‐Universität Erlangen‐Nürnberg (FAU) Erlangen Germany; ^2^ Comprehensive Cancer Center Erlangen EMN (CCC Erlangen‐EMN) Erlangen Germany

**Keywords:** brachytherapy, image guidance, needle navigation

## Abstract

**Background:**

Intraoperative cone‐beam computed tomography (CBCT) provides a valuable option for accurate three‐dimensional applicator positioning in gynecologic brachytherapy, but is associated with radiation exposure and increased intervention time especially in case of repeated CBCT imaging being required for creating a sufficient implant arrangement.

**Purpose:**

To reduce the need for multiple CBCT scans for corresponding applicator verification, this work proposes two methods for needle path navigation, including corrections of potential bending in situ, by combining infrared tracking with planar x‐ray imaging for enabling accurate intraoperative needle guidance.

**Methods:**

An examined 200 mm brachytherapy needle was rigidly mounted on an infrared‐reflective tracking tool to enable real time tracking. Two planar x‐ray images, acquired from varying distinct angles, were used to determine the exact 3D position of the needle tip region via backprojection. A spline was fitted through the obtained coordinates to reconstruct the full needle path. Based on this, only a single initial CBCT scan was required to visualize the predicted needle path within this scan. Additionally, a second approach for needle prediction was presented focusing on only one planar x‐ray image by incorporating prior needle bending information from the initial CBCT scan. Both methods were evaluated in preclinical studies and validated against a corresponding ground‐truth obtained from CBCT.

**Results:**

The proposed method considering two planar x‐ray images successfully reconstructed the needle path with deviations of less than 1 mm from the CBCT reference scan, when using at least 20° offset between the x‐ray image acquisitions. The single‐scan approach, using prior bending information, yielded promising results with deviations at the tip of below 1.3 mm.

**Conclusions:**

Both described methods demonstrated their feasibility in preclinical studies, showing potential to improve and accelerate clinical implantation workflows by means of needle navigation in the future.

## 1 INTRODUCTION

Accurate placement of applicators is essential in brachytherapy, making image‐based verification of three‐dimensional (3D) applicator positions in situ a critical component of the clinical workflow. In this respect, 3D image‐guided adaptive brachytherapy has demonstrated improvements over conventional two‐dimensional (2D) techniques, including reduced local recurrence and improved patient survival rates.^1^, ^2^, ^3^, ^4^ In current practice, ultrasound is commonly used for intraoperative needle guidance in brachytherapy for cervical cancer.^1^ However, in some cases, this imaging modality may not provide sufficient visualization of the applicators, especially for very deep‐seated or laterally extended tumors.^5^, ^6^ In a recent study, Karius et al.^7^ demonstrated significant improvements in quality and safety of gynecologic brachytherapy treatments when using intraoperative cone‐beam computed tomography (CBCT) in addition to ultrasound compared to sole ultrasound guidance. However, CBCT scans are associated with additional radiation exposure to patients, with typical weighted cone‐beam dose index (CBDI_w_) values of 6.7–11.8 mGy per 3D scan.^7^ Furthermore, the integration of CBCT in such workflows requires increased time for the interventional procedure, since the clinical staff has to leave the operating room for radiation protection reasons. As previously reported, corresponding CBCT imaging can prolong the overall intervention time by 28% (referring to 19 min) on average and up to 58% (referring to 44 min) for individual patients, especially in case of several CBCT scans being required to correct applicator positions.^7^

To address this issue, previous investigations^5^, ^8^ proposed a real time tracking method using infrared cameras, especially for tracking brachytherapy needles. By fixing needles to a tracking tool equipped with passive infrared markers, the respective needle course could be determined considering a fixed rigid offset between the markers and the needle. With this approach, the tip of a 200 mm rigid needle was predicted with an accuracy of 1.51 ± 0.76 mm based on tracking compared to direct reconstructions on CBCT scans. Only a single CBCT scan was thereby required for projecting the calculated needle path into the phantom's “anatomy”, enabling a reasonable needle verification with avoiding repetitive CBCT acquisitions.^5^

However, in actual clinical scenarios, inserting needles into the target volume may be associated with bending of the needles in situ, for example due to tissue resistance. This is of particular relevance, since needle bending occurring during implantations can compromise treatment quality by preventing the needle from reaching intended anatomical regions or achieving intended geometric configurations. As a result, the created implant geometry can be suboptimal, limiting the planner's ability to create an optimal dose distribution during subsequent postoperative treatment planning. This may lead to reduced target coverage, unbalanced dose distributions, or the need to accept higher doses to organs at risk (OARs) to compensate for unfavorable needle placement as well as to an increased risk for OAR perforations. In severe cases, as mentioned above, repositioning or removal of needles may be required, thus prolonging the intervention time. Recent examinations^9^ of cervical cancer cases in this respect reported intraoperative bending of up to 25.7 mm by comparing tip position of bent needles of 200–220 mm length to straight trajectories.

For this reason, taking the large extent of potential bending into account, infrared tracking alone was considered insufficient for accurately determining the entire path of the needles for real patient cases.^9^ Nevertheless, the latter is considered important especially for the treatment of cervical cancer patients with extensive vaginal involvement, where dwell positions of the afterloader source will have to be located at larger distances from the needle tip (that may have been shifted very deep into the pelvis for the treatment of the entire tumor) as well, rendering the knowledge of the full needle path—and not only of the needle tip—in relation to the anatomy crucial for creating a high‐quality implant. Combining tracking with the information about the extent of needle bending therefore aims to provide a suitable option for navigating brachytherapy implantations. In this respect, we present two approaches that enable path predictions. Our first method combined infrared tracking with two planar x‐ray images to reconstruct the entire 3D path of needles considering bending detected on the radiographs with different angular offsets. A second approach aims at the utilization of only one single planar x‐ray image and using preliminary bending information taken from the single CBCT scan necessarily acquired to display the patient's anatomy. Both techniques therefore aim to reduce radiation exposure and procedural time while realizing actual needle guidance. The description, assessment, and validation of the aforementioned approaches form the scope of the present work.

## 2 MATERIALS AND METHODS

### 2.1 Current clinical workflow

In our current clinical workflow, we use CBCT scans acquired with the mobile CBCT device ImagingRing m (medPhoton, Austria; for further technical descriptions, please refer to the following section) in combination with ultrasound for the verification of applicator positions in situ. As described in detail by Karius et al.,^7^ significant dosimetric improvements of implantations were achieved by applying this workflow compared to a previous workflow based on sole ultrasound guidance.

To be concrete, after the initial gynecological examinations in the lithotomy positions involving ultrasound imaging and palpation, the mobile CBCT system is moved over the patient to reach the imaging position. Afterward, the actual implantation of the intrauterine probe and potential interstitial needles starts by means of ultrasound guidance. Subsequently, a CBCT scan is acquired to validate the correct applicator positions in relation to the anatomy in 3D.^7^ This step is required, since the exact needle position can in some cases not be visualized using ultrasound alone (e.g., for very deep‐seated or laterally extended tumors),^5^, ^6^ as also mentioned in the introduction. For the CBCT imaging process, the patient legs have to be lowered a bit to allow for full gantry rotations, and the medical staff has to leave the intervention room for radiation protection reasons. After the CBCT scan has been acquired, the clinical staff reenters the operating room. Based on this scan, if the position of an applicator needs to be corrected, the applicator is adjusted and a further CBCT scan is acquired after the clinical staff again has left the room. This process is iteratively repeated until all desired applicator positions are reached, which can require multiple CBCT scans taken in succession depending on the clinical case. As drawback of this procedure increasing the safety and quality of implantations, the CBCT scans are associated with additional radiation exposure and the iterative process increases the overall intervention time.^7^ Therefore, the present work proposes the utilization of infrared tracking combined with planar x‐ray imaging to overcome the need for multiple CBCT scans to validate applicator positions, while enabling a navigated implantation, as described in detail in the following.

### 2.2 CBCT integrating infrared tracking

**FIGURE 1:**
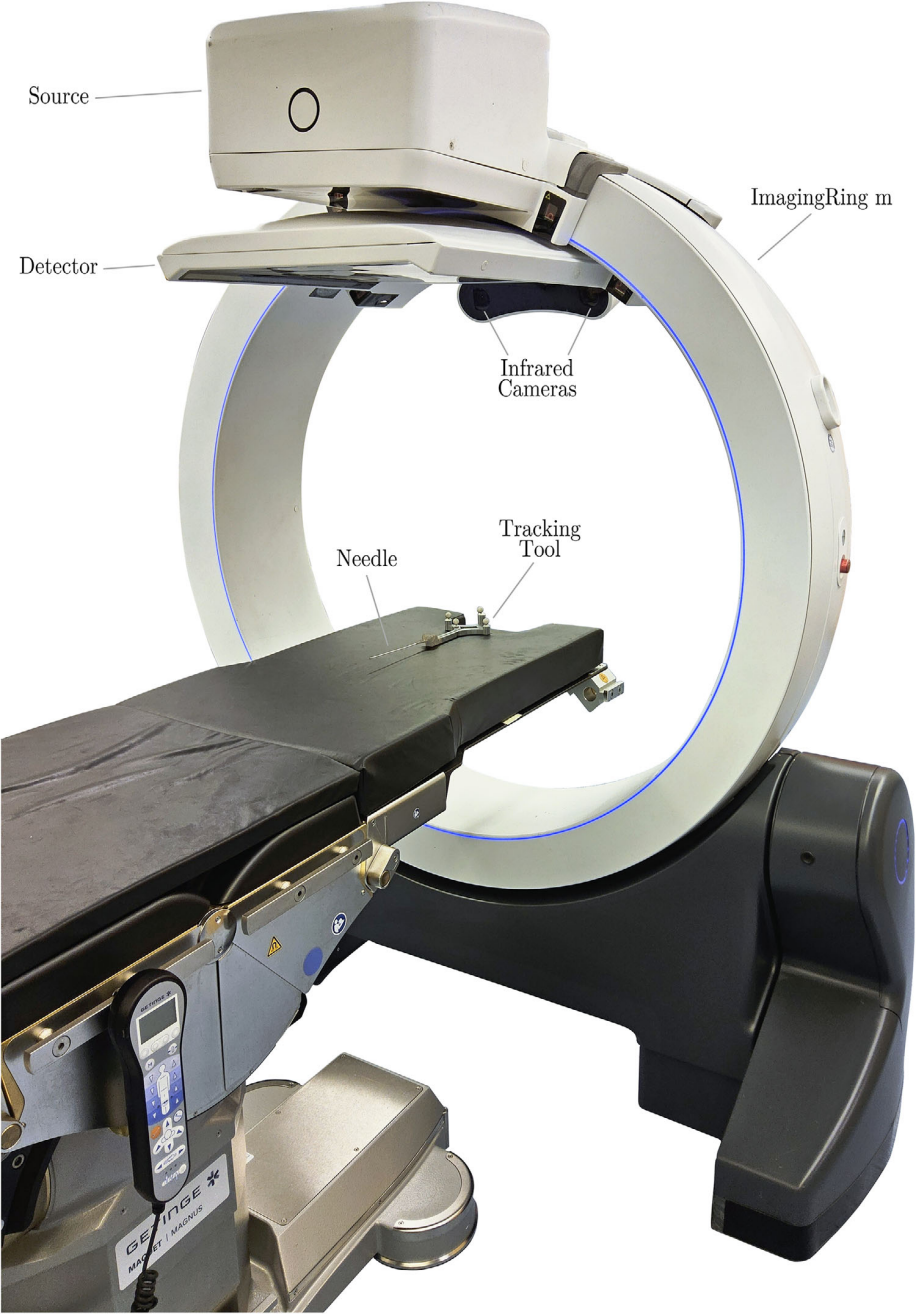
Experimental setup with the source and detector of the ImagingRing in their default positions. The tracking tool with the fixed needle was placed on the table, with the needle positioned in the scanning area and the markers within the field of view of the ImagingRing's infrared cameras.

To enable the combination of infrared tracking, CBCT scans, and planar x‐ray imaging integrated into one device, we utilized the mobile CBCT system ImagingRing m. Its gantry bore has a diameter of 121 cm with a source‐detector distance of 126 cm and a source‐axis distance of 74.3 cm. The corresponding flat‐panel detector of size 43.2×43.2 cm^2^ operated with a frame rate of 12 Hz features an active area of 2880×2880 pixels with 150 µm pixel pitch. For clinical scans and all experiments conducted in this work, a 2×2 detector binning was applied. Source and detector of the ImagingRing can rotate independently from each other around the gantry, enabling non‐isocentric as well as large field of view imaging.^10^ Motorized wheels and a battery mode allow for flexible movements of the ImagingRing, comprising lateral and longitudinal translations, rotations, and gantry tilts. Motions and imaging are controlled by a tablet PC connected via Wi‐Fi.^8^, ^11^ To enable infrared tracking, the device is equipped with two Prime^x^ 13 W infrared cameras (OptiTrack, USA),^12^ rigidly mounted at the top of the gantry (see Figure 1). With horizontal and vertical fields of view of 82° and 70°, respectively, the cameras are positioned to fully cover the CBCT scan area. Additional technical descriptions have been provided in detail previously regarding the ImagingRing, the tracking cameras, and their integration into a combined system, including registration and quality assurance procedures.^8^, ^11^, ^13^

### 2.3 Needle guidance using infrared tracking

**FIGURE 2:**
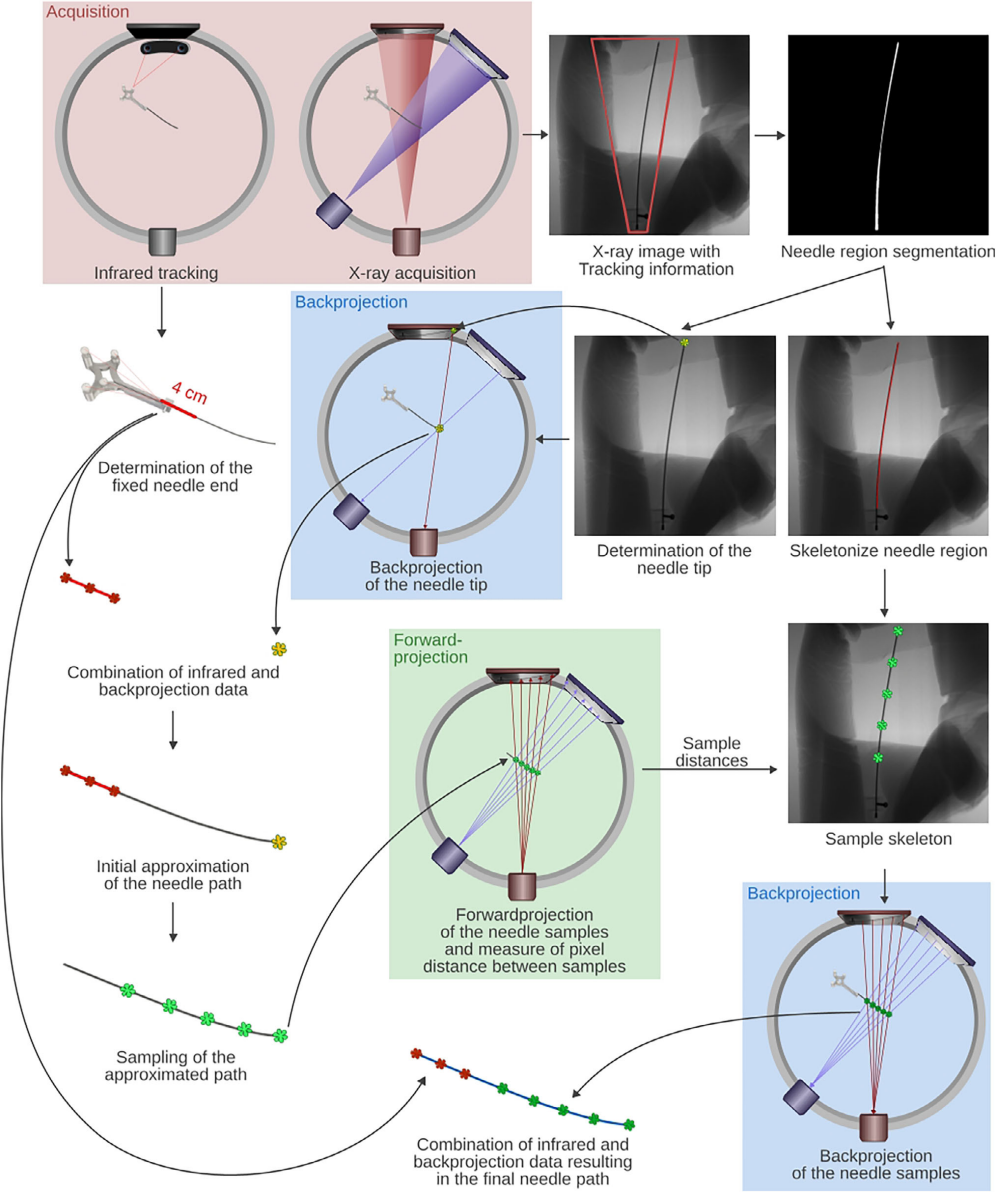
Workflow for needle path reconstruction using a combination of infrared tracking and limited angle back projection. First, infrared tracking was performed and two planar x‐ray images were acquired. The first 4 cm of the needle, fixed to the tracking tool and assumed to be rigid, were determined via infrared tracking. The needle region was segmented from the x‐ray scans within the determined needle region, and both the skeleton and the needle tip were extracted. The tip coordinates from both images were back projected to obtain the 3D coordinate of the needle tip. An initial needle path was estimated using the tracked needle region and the reconstructed tip, and sampled in 5 mm steps. These samples were forward projected onto the x‐ray detector to determine accurate sample distances, which were used to sample the needle skeleton. The corresponding samples were again back projected. Finally, a second‐order spline was fitted through the combined set of back projected and tracked samples resulting in the final 3D needle path.

**FIGURE 3:**
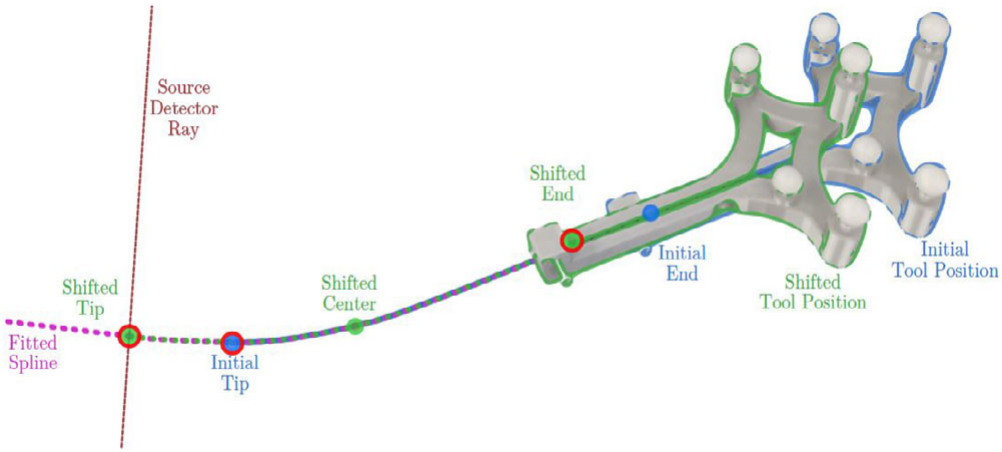
Illustration of the needle path reconstruction of a shifted needle, using prior information from a CBCT scan of the initial position (blue) and a single planar x‐ray image with infrared tracking data of the shifted position (green). The idea of this approach is to capture the trajectory of a needle including potential bending that has already been implanted to a certain depth once, and then insert the needle even deeper under radiography control. For each acquired planar x‐ray image (which could be recorded with a user‐selected frequency up to the detector frame rate of 12 Hz), the entire 3D needle path of the shifted needle can be calculated and output during implantation using this approach. The tip position of the shifted needle was determined by the intersection of the back projected tip coordinate from the planar x‐ray scan (red dashed line) and the fitted spline of the initial needle path (pink dashed line). The shifted needle path (green dashed line) was then reconstructed based on the circles marked in red.

The integration of infrared tracking into the ImagingRing aims to localize and predict the course of applicators within the CBCT coordinate system in real time.^5^ In this work, a 200 mm brachytherapy needle was used in all experiments. The needle was mounted on a 3D‐printed tracking tool that was designed in‐house and contained four passive infrared markers (see Figures 1, 2, 3). To establish the spatial relationship between the markers and the needle tip, a CT scan with 0.3 × 0.3 × 1 mm^3^ voxel size was acquired using a SOMATOM go.Open Pro CT scanner (Siemens Healthineers, Germany). From the CT scan, the 3D coordinates of the four markers and the needle tip were extracted. Based on this, the rigid transformation between the known (CT‐derived) marker configuration and the marker coordinates tracked by the infrared cameras was calculated. Applying this transformation also to the CT‐derived needle tip position yielded the tracked tip coordinate in the infrared tracking coordinate system. Since the infrared tracking system was calibrated with respect to the CBCT system, the tracking coordinates were aligned with the CBCT coordinates, enabling the visualization of the needle tip within the CBCT volume.^5^, ^8^ This method was extended to any point along the needle path, allowing the reconstruction of the entire path of a rigid needle based on infrared tracking data.

### 2.4 Needle path determination using x‐ray back projection

To enable needle navigation for brachytherapy implantations, our first approach utilized a combination of infrared tracking with two planar x‐ray scans. This procedure was chosen to address the lack of information about needle bending gainable from infrared tracking alone. Starting with the obvious fact that the entire 3D position of objects can be determined based on two orthogonal x‐ray projections, we investigated how small the angular offset between the two projections actually can be in order to still obtain a reasonable 3D position of the examined needle. This was required since a gantry rotation by 90° for obtaining orthogonal projects is a lasting procedure, making a smooth “real time” navigation impossible. However, reducing the angular offset between the projections for capturing needle bending in situ to only a few degrees could enable implantations during permanent small back and forth gantry rotations. Combined with infrared tracking, which already provides real time information about the needle course under the assumption of rigidity,^5^ this approach was established to provide near‐real time needle navigation.

To examine this approach, the whole workflow considered in this work is visualized in Figure 2. We adapted the needle tracking approach described in Section 2.3. to predict the four centimeter of the needle closest to its fixation in the tracking tool, assuming that this part of the needle was rigid based on our experience in handling the applicator‐tool assembly. The tracked needle path was then sampled in 5 mm steps for later steps. Additionally, two planar x‐ray images were acquired from distinct gantry angles. For instance, just to provide an example, the first image was acquired with a source angle of 0°, while the second one was acquired at a source angle of 20°, and the detector angles of 180° and 200°, respectively. The angles used for our analysis are presented in Section 2.5 in more detail.

In each case, we used the infrared tracking to determine the area of the x‐ray image where the needle, including potential bending, was expected to be. In clinical studies, this tracking information could be used to collimate the x‐ray image to further reduce the radiation dose to patients. Within the determined area, the needle path was segmented from the image background using a two‐stage Canny edge detection algorithm.^14^ First, edge detection was applied to the image rescaled to 1152 × 1152 pixels (40% of the original 2880 × 2880 pixels of the x‐ray image). This initial pass utilized a lower pixel intensity value threshold of 50, an upper threshold of 150, a 3 × 3 Sobel filter, and the L1 gradient norm. In a second step, the thresholds were adjusted relative to the maximum pixel intensity (20% for the lower and 60% for the upper threshold), and the detection was performed on the full‐resolution image (2880 × 2880 pixels). The final edge image was created considering the union of edge pixels from both detection runs. Afterward, morphological dilation and closing to adjust the line width for subsequent processing steps was performed. The Probabilistic Hough Transform^15^ was subsequently employed to robustly identify and retain the correct needle edges. Lines were detected with a distance resolution of one pixel and an angular resolution of 0.5°. Initial filtering rejected lines shorter than 50 points or those containing gaps exceeding 20 pixels. Further refinement was achieved by discarding lines with incorrect orientations, determined via forward projection of the infrared‐tracked needle onto the image plane. The remaining lines whose angles differed by less than 20° were then merged. Additional morphological closing was performed to fill the entire needle region. Since the resulting region was still imprecise due to the finite edge width, intensity thresholding was applied to the x‐ray image within the detected needle region, yielding the final segmented needle path. Based on these segmentations, the needle tip was determined in each of both images, and subsequently backprojected to obtain its 3D position within the CBCT scanner's coordinate system. For this backprojection, to compute the 3D tip position from the 2D pixel coordinate in each x‐ray image, the pixel spacing of the detector as well as its spatial position and orientation were taken into account. Considering the x‐ray image i, the ray from the source position $s_{i}$ to the corresponding needle tip coordinate $x_{i}$ on the detector plane in 3D space was calculated and normalized:

(1) $$\;\hat{e_{i}}=\frac{x_{i}-s_{i}}{\left|\left|x_{i}-s_{i}\right|\right|}\;$$

Uncertainties, for example, in pixel selection, prevent the rays from intersecting at a single point. Therefore, no exact analytical intersection could be determined,^16^ and an approximation was used instead. The 3D coordinate with the smallest distance to both rays was calculated by first computing the normal vector n to both rays as the cross product of the ray directions:

(2) $$n=\hat{r_{1}}\times \hat{r_{2}}\;$$

Using n, the points $p_{1}$ and $p_{2}$ on each ray were calculated as closest point to the other ray:

(3) $$\;p_{1}=s_{1}+t_{1}\cdot \hat{r_{1}}\;with\;t_{1}=\frac{\left(\hat{r_{2}}\;\times n\right)\cdot \left(s_{2}-s_{1}\right)}{n\cdot n}\;$$

(4) $$\;p_{2}=s_{2}+t_{2}\cdot \hat{r_{2}}\;with\;t_{2}=\frac{\left(\hat{r_{1}}\;\times n\right)\cdot \left(s_{2}-s_{1}\right)}{n\cdot n}$$

The final 3D tip position c was then determined as the midpoint between $p_{1}$ and $p_{2}$:

(5) $$c=\frac{p_{1}+p_{2}}{2}\;$$

In the next step, a second‐order spline was fitted to the sample points obtained from the tracking data and the backprojected tip position, providing an initial approximation of the entire needle path in the 3D CBCT coordinate system. The needle tip and end are based on observed data from the backprojection and infrared tracking, whereas the central section was interpolated.

To refine these interpolated sections with measured data, further coordinates along the needle path were calculated in 5 mm steps using backprojection from the x‐ray images. Because oblique needle orientations lead to nonuniform spacing of these samples in the detector plane, corresponding 2D sampling points for the backprojection had to be calculated first. For this purpose, the approximated 3D needle path was sampled at 5 mm intervals starting from the tip. Each sampling point was forward projected from both x‐ray sources onto the respective detector planes, and the intersection points were determined. The distances between consecutive intersection points defined the sampling intervals in the 2D images, after conversion into pixel units using the detector's pixel spacing. Based on these distances, the needle paths in both images were sampled along their skeletonized needle regions. Assigning indices to the samples, starting with 0 at the needle tip, allowed unambiguous correspondence of samples between the two x‐ray images. The matched 2D samples were then backprojected into 3D space as described above. Finally, the resulting 3D points from both the tracking data and the x‐ray image‐based backprojection were merged, ordered along the needle axis, and fitted with a second‐order spline. This yielded the reconstructed 3D needle path with reduced uncertainty compared to the initial approximation.

### 2.5 Analysis of the needle path determination using x‐ray back projection

To evaluate the accuracy of the needle path reconstruction using the approach described above, a 200 mm brachytherapy metal needle was positioned in air, with the tracking tool placed on the proximal end of the needle. This positioning was consistent with the implantation direction applied in clinical scenarios, as shown in Figure 1.

To investigate the influence of the angular relationship between the x‐ray scans, reconstructions were performed using x‐ray images acquired at different gantry positions. Eleven scans with source angle positions from 0° to 20° in 2° steps were acquired considering one fixed needle trajectory (the detector was always positioned opposite the source) with simultaneous infrared tracking. Out of these images, all possible combinations of two scans with angular differences ranging from 2° to 10° in 2° steps were selected for performing the described backprojection approach to obtain a 3D needle course. To provide an example, for investigating the angular differences of 10°, we considered the x‐ray image pairs acquired at source angles of 0° and 10° as well as of 2°and 12°, and so on. Additionally, 25 scans were acquired considering a second fixed needle trajectory with source angles from 0° to 120° in 5° steps. From these scans, scan pairs with angular differences ranging from 5° to 90° in 5° increments were formed, and the 3D needle course was calculated for each pair. Selecting a maximal acquisition angle of 120° ensured multiple backprojections from orthogonal acquisition angles.

To obtain a ground‐truth of the entire 3D needle path, a CBCT reference scan was acquired, from which the needle path was extracted by thresholding the volume and sampled beginning from the tip position with the same sampling distance as in the backprojection step. This ground‐truth needle path was then compared to the needle path obtained by the approach combining tracking and two radiography images. For this comparison, Euclidean distances were evaluated at three positions along the needle. First, the distance between the two corresponding needle tip positions was calculated. Second, the distance between the two corresponding end positions of the needle was determined. Third, the central accuracy was assessed by defining a section comprising half of the total needle length centered around the midpoint, for which the mean Euclidean distance across all sampled points was calculated.

### 2.6 Needle path determination using prior information

While determining needle courses based on the aforementioned method utilizing two planar x‐ray images with a respective angular offset can be used to detect bending in three dimensions, it still requires some acquisition time due to the associated gantry rotation. For this reason, we also examined a second approach for needle navigation focusing on a combination of prior knowledge about bending detected on an initial CBCT scan, infrared tracking of the needle end, and only one single planar x‐ray image taken from a stationary gantry angle. It was therefore examined if the missing bending information along the third spatial dimension associated with the acquisition of only one two‐dimension planar x‐ray image can be compensated for by considering the aforementioned prior information, which could also allow for a real time navigation in case of x‐ray images being acquired in continuous fluoroscopy mode.

The proposed method visualized by Figure 3 started with the acquisition of a single CBCT scan at a time point when needles have already been implanted to a specific user dependent depth. From this scan, the initial needle path was extracted by thresholding the volume and interpolated between the CBCT slices by means of a second‐order spline. If the needle was shifted, i.e. inserted deeper into the phantom or body, it was assumed to be shifted along this defined path. For the process of navigation, infrared tracking was used to determine the updated position of the end of the needle and, hence, the shifting length. Additionally, a single planar x‐ray scan was acquired with opposing source and detector positions. From this image, the needle tip was extracted as mentioned in the first presented method and back projected from its detector plane position toward the source position along the direction vector. The new tip position of the shifted needle was then determined by calculating the pair of points, one on the back projection ray $r(t_{r})$ and one on the previously fitted and extrapolated spline $s(t_{s})$, that were closest to each other in 3D space. The back projection ray was parameterized as:

(6) $$r\;\left(t_{r}\right)=\left(x_{\mathrm{tip}}+t_{r}\cdot \hat{r}\right)\;$$

where $\left(x_{\mathrm{tip}}\right)$ denotes the 3D coordinate of the tip pixel, $\hat{r}$ the normalized ray direction from $\left(x_{\mathrm{tip}}\right)$ to the source, and $t_{r}$ the ray parameter. The spline $s(t_{s})$ was defined by the interpolation function from the scipy.interpolate.^17^ The parameters $t_{r}$ and $t_{s}$ were jointly optimized by minimizing the Euclidean distance.

(7) $$\min_{t_{r},t_{s}}\left|\left|s\left(t_{s}\right)-r\left(t_{r}\right)\right|\right|\;$$

The final needle tip position *c* was computed as the midpoint of the line segment connecting the closest points on the ray and the spline:

(8) $$c=\frac{r\left(t_{\mathrm{optim}}\right)+s\left(t_{\mathrm{optim}}\right)}{2}$$

Finally, the resulting 3D needle path was determined by fitting a second‐order spline through the initial needle tip position determined from the CBCT scan, the updated tip position and the distal needle position determined directly via the infrared tracking, yielding the final reconstructed needle path.

### 2.7 Analysis of the needle path determination using prior information

To analyze the needle reconstruction using prior information, two insertions were performed using both a straight and a bent needle. This is, since in contrast to the backprojection approach described in Section 2.4, using only a single x‐ray image for reconstruction in addition to prior bending‐information may be associated with higher uncertainties in needle path determination, especially when bending occurs within the plane running parallel to the backprojection ray. By evaluating both the straight and bent path (for the latter case, the needle tip deviated by 28.0 mm from the straight position), we aimed to investigate whether the needle can still be fully reconstructed even when the needle trajectory deviated from a purely one‐dimensional shift. The experiments investigating the prior‐information approach thus considered a bent needle as well, whereas no bent needle was examined with the previous approach described in Section 2.4/2.5 (as the backprojection method inherently accounts for bending through considering the two acquired planar scans).

To assess the prior‐information approach, the corresponding needle was inserted into a gel pillow, and a CBCT scan was acquired from which the initial needle path was determined. The respective needle was then shifted by 27.9 mm for the straight and by 10.0 mm for the bent needle, and an x‐ray scan for determining the needle tip of the shifted needle was acquired. To examine the potential effect of angular dependencies, several x‐ray scans with source angles ranging from −20° to 20° in 5° increments (with opposite detector positions) were taken, resulting in nine scans used for separate calculations of the same needle path according to the descriptions provided in Section 2.6. Simultaneous infrared tracking to determine the needle end position was conducted in each case, as described above. To validate the needle path as reconstructed by this approach, CBCT scans were acquired after the shifting process to serve as a reference, from which the actually implanted needle path was determined. The Euclidean distances between the reconstructed path and the reference path were measured at the needle tip, center, and needle end as for the previous approach.

## 3 RESULTS

### 3.1 Needle path determination using x‐ray back projection

**FIGURE 4:**
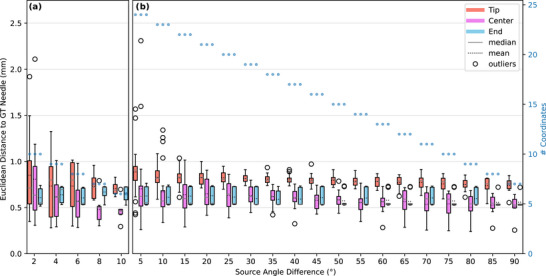
Analysis of the Euclidean distances between the reconstructed needle and the CBCT reference (ground truth; GT), depending on the varying angles between the planar x‐ray images. The Euclidean distances measured at the needle tip, center region, and needle end are shown on the left *y*‐axis. The second (right) *y*‐axis indicates the number of reconstructed coordinates (i.e., samples) included in each boxplot. This number decreases with increasing angular difference, since fewer image pairs could be formed from the available input scans, as described in Section 2.5. The *x*‐axis shows source angle differences between the paired scans used for back projection. Subplot a) focuses on the needle trajectory investigated using small angular differences ranging from 2° to 10° (2° steps) and subplot b) shows results for the second needle trajectory using a broader range of source angle differences from 5° to 90° (5° increments). Please note that the boxplots used in this diagram serve only to visualize the needle reconstruction deviations rather than infer statistical significance, and that the data obtained from needle tip, center, and end originating from fixed needle trajectories are not statistically independent.

Figure 4 shows the Euclidean distances between the reconstructed needle paths and the needle path extracted from a CBCT reference scan. For reconstructing the needle path using the presented approach, various angular differences between the source positions were analyzed. As all possible x‐ray images with the desired angular differences were paired, a higher amount of pairs existed for smaller angles than for larger angles.

For the source angle difference of 2°, the tip coordinate reconstructed from two x‐ray images deviated by up to 1.92 mm (minimum deviation at 0.35 mm) from the corresponding one determined directly via CBCT imaging with a mean deviation of 0.85 ± 0.48 mm. The center region of the needle showed deviations up to 2.11 mm with a mean of 0.81 ± 0.51 mm (minimum deviation at 0.31 mm). For the 5° difference, the maximum deviation in the tip region was slightly reduced to 1.57 mm, with the mean remaining at 0.89 ± 0.26 mm, while the center region showed a maximum deviation of 2.31 mm with a mean of 0.78 ± 0.57 mm. As the angular difference increased, the deviations of the reconstructed tip position and center region from the corresponding reference scan values decreased, as described below. However, the needle end demonstrated constant results across all angular differences, with maximum deviations consistently below 0.75 mm as well as mean deviations between 0.56 ± 0.06 mm and 0.63 ± 0.10 mm.

The maximum deviations at the tip, center, and end of the needle remained below 1.34 mm for an angular difference between source positions of 10° and above, with mean deviations below 0.83 ± 0.11 mm. For angular differences of 20° and higher, the maximum deviations decreased further and remained consistently below 1 mm (mean deviations at or below 0.82 ± 0.08 mm). This trend clearly indicated that angular differences of at least 20° had a positive effect on the needle path determination. Increasing the angle beyond 20° up to 90° did not result in notable improvement at the needle tip (mean deviation decreases from 0.82 ± 0.08 mm to 0.73 ± 0.09 mm) and the center region (from 0.65 ± 0.15 mm to 0.51 ± 0.12 mm).

### 3.2 Needle path determination using prior information

**FIGURE 5:**
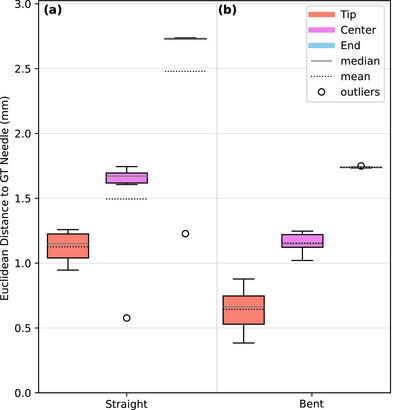
Analysis of the needle reconstruction using prior information. The graph shows the deviations to the reference (ground truth; GT) needle path originating from one simulated shift of a straight needle of 27.9 mm (a) and one simulated shift of a bent needle of 10 mm (b). For both needles, the Euclidean distances between the reconstructed needle using our suggested approach and the needle extracted from a CBCT reference scan are shown on the *y*‐axis (measured at the needle tip, center region, and needle end). Please note that the boxplots used in this diagram serve only to visualize the needle reconstruction deviations rather than infer statistical significance, and that the data obtained from needle tip, center, and end originating from fixed needle trajectories are not statistically independent.

Figure 5 shows the reconstruction results for the shifted straight and bent needle in a gel pillow. The deviations at the needle tip, center, and needle end are visualized as Euclidean distances from the reference needle path obtained from a CBCT scan in the shifted position. The graph presents results for the reconstructions of the straight needle which was shifted by 27.9 mm (Figure 5a), and for the bent needle which was shifted by 10.0 mm (Figure 5b). For the straight needle, the successfully reconstructed paths showed a mean deviation of 1.12 ± 0.12 mm at the tip (maximum deviation of 1.26 mm), whereas the center region showed larger mean deviations of 1.49 ± 0.41 mm (maximum deviation of 1.74 mm). The reconstruction of the bent needle achieved lower deviations per needle region, with a mean tip deviation of 0.64 ± 0.15 mm (maximum deviation of 0.88 mm), and a center deviation of 1.16 ± 0.07 mm (maximum deviation of 1.25 mm).

## 4 DISCUSSION

We developed two approaches that aim to reduce radiation exposure and overall treatment time during gynecological brachytherapy by limiting imaging to a single CBCT scan and, at most, two additional planar x‐ray acquisitions per needle repositioning, instead of repeated CBCT scans. At the same time, visualization of the applicators in situ with respect to the anatomy is preserved, as the reconstructed needle path can be visualized within the initially acquired CBCT scan.

In the limited angle backprojection algorithm, we analyzed the reduction of the angular offset between two x‐ray images used for backprojecting the needle path. Reducing the angular offset would accelerate the workflow during brachytherapy needle implantation as the source and detector require less time to rotate between the scan positions for the backprojection. A minimal angular offset of only a few degrees could allow for needle guidance with near real time performance. However, reducing the angular difference results in less accurate backprojection. Ideally, an angular difference of 90° should be used, as it minimizes the geometric uncertainty of triangulating 3D coordinates.^16^ Nonetheless, the results presented in Figure 4 demonstrate that source angle differences of at least 20°, with the detector being in opposing position (180° source‐detector angle), are sufficient to achieve a reconstruction accuracy comparable to that obtained with a 90° offset. The maximum deviations remained below 1 mm when using an angular offset of at least 20°, whereas for smaller offsets they increased to values of up to 2.31 mm. These results can be attributed to the backprojection becoming more susceptible to uncertainties when performed from small angles. Based on these findings, we recommend using angular offsets of at least 20° to minimize errors caused by triangulation uncertainties. It is also worth noting that the accuracy of the needle end coordinate remains largely unaffected by the angular configuration. This is because the end position was derived from the infrared tracking system, rather than the x‐ray reconstruction. As a result, the accuracy of the needle end remains consistent across all evaluated source angle differences.

Please note that the novelty of the aforementioned approach does not lie in the utilization of radiography images for the intended purpose per se, but in the integration of limited‐angle planar imaging with infrared tracking technology rigidly mounted and intrinsically registered to our mobile x‐ray system. This solution enables in particular reconstructions of the full needle trajectory including potential bending in situ for allowing a direct visualization of the reconstructed path within an initially acquired CBCT scan without additional registration steps being required. To the best of our knowledge, considering this unique combination for bending correction has not been reported previously within the context of intraoperative navigation for interstitial brachytherapy, but may represent (based on the results of our conducted feasibility study) a promising solution to address corresponding clinical issues in the future.

The evaluation of the needle path determination using prior information indicates that sub‐millimeter accuracy can be achieved, as demonstrated in the case of the bent needle, which exhibited a smaller shift than the straight needle. For both the straight and bent needle setups, the deviation between the reconstructed needle and the reference needle was slightly larger in the central region, and especially at the needle end, than at the tip. The higher deviations at the needle end were primarily caused by uncertainties in the infrared tracking data, which directly determine the distal needle position. In contrast, the tip position benefits from radiographic information, explaining the higher accuracy reachable for this part of the needle trajectory. The approach achieved a maximal accuracy at the tip of up to 0.38 mm using the bent needle which was shifted by 10.0 mm from the initial position. The straight needle, which was shifted further compared to the bent needle by 27.9 mm from the initial position, showed larger mean Euclidean distances at every needle region. The lower error observed for the bent needle can therefore be attributed to the smaller applied shift and the fact that the needle trajectory information from the prior CBCT remained valid after repositioning to a larger extent than for the straight needle, where even small deviations from the exact straight path associated with the much deeper implantation into the gel pillow could have led to the increased deviations observed. These results indicate that for larger shifts, this approach may be too imprecise as the needle bending after the correction may differ from the initial one. Distance limits regarding this issue could not be determined in the present work, but are subject to further investigations beyond the feasibility analysis performed in this study. In contrast, small needle corrections may not affect the bending and could be tracked in real time using this approach. This is particularly valuable in clinical settings, where radiation dose should be minimized and workflows could be accelerated by avoiding additional scans. In this regard, both presented approaches provide clear evidence of their feasibility and potential to enable accurate needle path reconstruction through the combination of infrared tracking and CBCT as well as planar radiography.

Considering the results obtained with both proposed methods, it has to be mentioned that the current validation was limited to moderate needle bending and relatively small shifts, which do not entirely capture the full complexity of clinical implantations. In particular, larger or dynamically evolving bending may results in nonuniform curvature that may potentially not be reliably inferred using the presented approaches, especially using the navigation based on prior‐information. Future investigations will therefore have to extend the validation in several directions. First, controlled phantom experiments will have to be conducted in which bending magnitude, curvature profile, and deviation are systematically varied using predefined insertion angles to induce clinically relevant bending patterns. This will allow quantitative assessments of navigation accuracy as a function of bending magnitude. Second, repeated insertions under identical conditions will have to be performed to evaluate the variability and robustness of the proposed approaches. Third, scenarios with progressive bending along the insertion path will have to be investigated to assess the corresponding navigation performance under nonstatic bending conditions. These studies will provide a more comprehensive validation going beyond the feasibility‐driven design reported in the present work under conditions more closely resembling clinical practice.

Nevertheless, both proposed approaches have the potential to remarkably reduce procedure time in gynecological brachytherapy treatments. Unlike during CBCT acquisitions, the clinical staff could stay in the operating room during planar x‐ray imaging provided they wore appropriate lead aprons, eliminating delays associated with repeatedly leaving and reentering the room. Karius et al. recently reported^7^ an average time requirement of 11.9 min for one implant iteration based on CBCT imaging, which could be reduced up to the x‐ray acquisition time of a few milliseconds to seconds if needle navigation based on the proposed techniques is applied instead. Moreover, CBCT acquisitions require full gantry rotations, whereas the first proposed approach required only limited rotation and the second approach required none at all, further reducing procedural time. For the x‐ray back projection approach based on two planar x‐ray images, gantry angles could be selected to avoid collisions with the patient's legs, potentially eliminating the need to lower them during image acquisition. In the single x‐ray scan approach, leg repositioning could be avoided entirely as no gantry movement is performed. A further important aspect is the potential to considerably reduce patient radiation exposure, since the proposed methods require at most two planar x‐ray scans, one for the prior‐information approach and two for the backprojection approach based on two planar x‐ray images. In this respect, it has to be mentioned that single intraoperative CBCT scans acquired with the ImagingRing for gynecologic brachytherapy feature typical weighted cone‐beam dose index (CBDI_w_) values of 6.7–11.8 mGy,^7^ whereas a detector entrance dose of only 2.8 µGy has been reported for single planar x‐ray images of the pelvic region with the same device.^18^ Thus, the cumulative procedural dose considering repetitive CBCT imaging with two to four CBCT scans^7^ would be more than a thousand times larger than the dose resulting from even multiple planar x‐ray acquisitions potentially required for implant creation. Additionally, the irradiated area could be further minimized through collimation guided by the infrared tracking data for both approaches. By forwardprojecting the tracked needle onto the detector plane, the x‐ray beam could be restricted to the region where the needle is expected to appear, while including a margin to account for possible needle bending.

To the best of our knowledge, we are the first to propose the use of infrared tracking in combination with x‐ray imaging for needle navigation—including in situ needle deflection correction—in interstitial brachytherapy. While similar concepts^19^, ^20^, ^21^, ^22^ employing either standalone IR tracking^19^ or electromagnetic tracking have been described in the literature, these approaches share a common limitation: they rely on external cameras or field generators, which necessitate additional registration steps between imaging and tracking data. In contrast, our approach integrates the tracking components directly into the mobile CBCT system via rigid mounting. This eliminates the need for separate registration, potentially reducing navigation uncertainties—as previously discussed by Karius et al.^8^ Moreover, existing methods generally do not account for in situ needle deflection, nor do they enable full reconstruction of the entire needle trajectory. Our proposed technique, based on the fusion of infrared tracking and radiographic data—or alternatively, deflection information derived from a priori CBCT scans—allows for comprehensive in situ reconstruction of the entire needle path during the insertion process. We believe this may enable a more accurate and robust guidance during implantation, ultimately improving both the precision and safety of interstitial brachytherapy procedures.

To date, both presented methods had only been validated in preclinical experiments considering an air or gel phantom environment, which do not capture the complexity of human anatomy. So far, their application in clinical scenarios, in which additional factors such as reduced soft tissue contrast, overlapping anatomical structures, more complex needle trajectories, image noise, and the presence of multiple applicators may challenge the accuracy and reliability of needle detections in the planar x‐ray images, and hence also the accuracy of the entire navigation approach, is missing. To address these limitations, we plan to conduct phantom experiments also incorporating anatomical surrogates such as tissue‐mimicking phantoms including bony structures and multiple applicators to simulate clinically realistic imaging conditions. However, following these procedures, the actual clinical feasibility will only reveal from corresponding clinical feasibility trials to be performed under controlled conditions, i.e., by attaching the infrared‐tracking tool to already implanted applicators and thus retrospectively evaluating the accuracy of needle predictions compared to their actual positions obtained from intraoperative CBCT scans. These staged evaluations will help to identify potential failure modes and optimize the proposed approaches prior to a prospective utilization for needle navigation. Furthermore, an intended bending of the needle is sometimes induced when inserting the applicator and adjusting its positions to reach a specific target position. For the approach using prior information, this could cause a deviation of the spline which would result in incorrect needle path calculation as we assumed that the needle is shifted along the spline. In contrast, the first approach, in which the needle path is determined via backprojection from two different angles, appeared promising in determining precise needle courses even if the needle curvature deviated during shifting the needle. The applicability of both approaches should be assessed in further studies with phantoms and subsequently with clinical x‐ray images. While the focus of our investigations is particularly on the brachytherapy of cervical cancer, we are confident that applicable use cases arise for the treatment of other entities as well, as for instance for permanent^23^ or temporary^24^ prostate or breast^25^ brachytherapy implantation procedures.

Finally, while deviations between reconstructed needle paths and the actual needle trajectories obtained from CBCT reference scans are reported throughout the manuscript, the statistical power of our study is inherently limited by its feasibility‐driven design. The number of needle paths considered for each performed experiment was restricted, and, hence, individual measurements cannot be considered fully independent due to shared experimental conditions and the repeated use of the same setup. Consequently, formal hypothesis testing and statistical analyses were not performed, as such tests would risk overinterpretation of exploratory data. Instead, the reported results are intended to provide descriptive insights into feasibility of our approaches to correct needle bending. Statistical comparisons will be essential in future studies with larger, independent sample size to allow robust quantitative conclusions and generalizability of our findings.

## 5 CONCLUSION

We presented two approaches for needle guidance during brachytherapy. In the first approach, the needle path was determined by combining two planar x‐ray images with infrared tracking, and the resulting 3D path was visualized in a single CBCT scan. In the second approach, curvature information obtained from an initial CBCT scan during needle placement was used to reduce subsequent imaging to a single planar x‐ray acquisition. The feasibility of both approaches was evaluated in preclinical studies. The x‐ray back projection method achieved sub‐millimeter accuracy when using an offset of at least 20° between the x‐ray images, while the prior‐information method yielded accuracies of at least 1.3 mm around the needle tip. Overall, the feasibility of both approaches was successfully demonstrated, showing potential to accelerate clinical implantation workflows while reducing radiation exposure to patients. Further investigations are needed to analyze the robustness and accuracy of the approaches in clinical scenarios.

## AUTHOR CONTRIBUTIONS

All authors contributed significantly to the performed work and approved the final version of the manuscript to be published.

## CONFLICT OF INTEREST STATEMENT

The University Hospital Erlangen has research agreements with medPhoton and Elekta regarding ImagingRing m applications in brachytherapy.

## Data Availability

Data are provided by the corresponding author upon reasonable request.

## References

[1] Pieters B , Paulsen‐Hellebust T , Pieters B , Troost E . Image‐Guided Adaptive Brachytherapy. In: Troost E , ed. Image‐Guided High‐Precision Radiotherapy. Springer; 2022:179‐200. doi:10.1007/978‐3‐031‐08601‐4

[2] Lim YK , Kim D . Brachytherapy: a comprehensive review. Prog Med Phys. 2021;21(2):25‐39. doi:10.14316/pmp.2021.32.2.25

[3] Eustace N , Liu J , Ladbury C , et al. Current status and future directions of image‐guided adaptive brachytherapy for locally advanced cervical cancer. Cancers. 2024;16(5):1031. doi:10.3390/cancers16051031 38473388 10.3390/cancers16051031 PMC10931056

[4] Li F , Shi D , Bu M , Lu S , Zhao H . Four‐dimensional image‐guided adaptive brachytherapy for cervical cancer: a systematic review and meta‐regression analysis. Front Oncol. 2022;12:870570. doi:10.3389/fonc.2022.870570 35860574 10.3389/fonc.2022.870570 PMC9291247

[5] Karius A , Leifeld LM , Strnad V , Schweizer C , Fietkau R , Bert C . Initial needle tracking with the first standalone combined infrared camera—CT system for brachytherapy‐analysis of tracking accuracy and uncertainties. Strahlenther Onkol. 2025;201(2):163‐172. doi:10.1007/s00066‐024‐02253‐3 38967820 10.1007/s00066-024-02253-3 PMC11754369

[6] Knoth J , Nesvacil N , Sturdza A , et al. Toward 3D‐TRUS image‐guided interstitial brachytherapy for cervical cancer. Brachytherapy. 2022;21(2):186‐192. doi:10.1016/j.brachy.2021.10.005 34876361 10.1016/j.brachy.2021.10.005

[7] Karius A , Strnad V , Bert C , Fietkau R , Merten R , Schweizer C . Establishing an intraoperative, mobile CBCT‐based workflow for gynecologic brachytherapy: primary experience and benefit assessment. Front Oncol. 2025;15:1562670. doi:10.3389/fonc.2025.1562670 40308506 10.3389/fonc.2025.1562670 PMC12040815

[8] Karius A , Leifeld LM , Strnad V , Fietkau R , Bert C . First implementation of an innovative infra‐red camera system integrated into a mobile CBCT scanner for applicator tracking in brachytherapy‐initial performance characterization. J Appl Clin Med Phys. 2024;25(7):e14364. doi:10.1002/acm2.14364 38626753 10.1002/acm2.14364 PMC11244686

[9] Karius A , Strnad V , Lotter M , et al. Assessment of needle bending and tracking requirements for optimized needle placement in combined intracavitary/interstitial gynecologic brachytherapy. Strahlenther Onkol. 2026;202:40–51. doi:10.1007/s00066‐025‐02367‐2 39915305 10.1007/s00066-025-02367-2 PMC12819550

[10] Karius A , Szkitsak J , Strnad V , Fietkau R , Bert C . Cone‐beam CT imaging with laterally enlarged field of view based on independently movable source and detector. Med Phys. 2023;50(8):5135‐5149. doi:10.1002/mp.16463 37194354 10.1002/mp.16463

[11] Karius A , Karolczak M , Strnad V , Bert C . Technical evaluation of the cone‐beam computed tomography imaging performance of a novel, mobile, gantry‐based x‐ray system for brachytherapy. J Appl Clin Med Phys. 2022;23(2):e13501. doi:10.1002/acm2.13501 34905285 10.1002/acm2.13501 PMC8833290

[12] OptiTrack. Prime 13 W. Link: (accessed 09/30/2025). https://optitrack.com/cameras/prime‐13w/

[13] Karius A , Strnad V , Lotter M , Kreppner S , Bert C . First clinical experience with a novel, mobile cone‐beam CT system for treatment quality assurance in brachytherapy. Strahlenther Onkol. 2022;198(6):573‐581. doi:10.1007/s00066‐022‐01912‐7 35278094 10.1007/s00066-022-01912-7 PMC9165284

[14] Canny J . A computational approach to edge detection. IEEE Trans Pattern Anal Mach Intell. 1986;8(6):679‐698. doi:10.1109/TPAMI.1986.4767851 21869365

[15] OpenCV Foundation: Feature Detection—OpenCV 3.4 Documentation. Link: (accessed at 12/15/2025). https://docs.opencv.org/3.4/dd/d1a/group__imgproc__feature.html#ga8618180a5948286384e3b7ca02f6feeb

[16] Munn SM , Pelz JB . FixTag: an algorithm for identifying and tagging fixations to simplify the analysis of data collected by portable eye trackers. ACM Trans Appl Percept. 2009;6(3):1‐25. doi:10.1145/1577755.1577759

[17] Virtanen P , Gommers R , Oliphant TE , et al. SciPy 1.0: fundamental algorithms for scientific computing in Python. Nature Methods. 2020;17:261‐272. doi:10.1038/s41592‐019‐0686‐2 32015543 10.1038/s41592-019-0686-2 PMC7056644

[18] Karius A , Szkitsak J , Boronikolas V , Fietkau R , Bert C . Quality assurance and long‐term stability of a novel 3‐in‐1 x‐ray system for brachytherapy. J Appl Clin Med Phys. 2022, 23(9):e13727. doi:10.1002/acm2.13727 35848090 10.1002/acm2.13727 PMC9512339

[19] Auer T , Hensler E , Eichberger P , et al. 3D navigation for interstitial stereotaxic brachytherapy. Strahlenther Onkol. 1998;174(2):82‐87. doi:10.1007/BF03038480 10.1007/BF03038480 9487370

[20] Zhou Z , Jiang S , Yang Z , Xu B , Jiang B . Surgical navigation system for brachytherapy based on mixed reality using a novel stereo registration method. Virtual Reality. 2021;25:975‐984. doi:10.1007/s10055‐021‐00503‐8

[21] Strassmann G , Kolotas C , Heyd R , et al. Navigation system for interstitial brachytherapy. Radiother Oncol. 2000;56(1):49‐57. doi:10.1016/s0167‐8140(00)00209‐7 10869755 10.1016/s0167-8140(00)00209-7

[22] Wang W , Viswanathan AN , Damato AL , et al. Evaluation of an active magnetic resonance tracking system for interstitial brachytherapy. Med Phys. 2015;42(12):7114‐7121. doi:10.1118/1.4935535 26632065 10.1118/1.4935535 PMC4662673

[23] Karius A , Schweizer C , Strnad V , et al. Seed‐displacements in the immediate post‐implant phase in permanent prostate brachytherapy. Radiother Oncol. 2023;183:109590. doi:10.1016/j.radonc.2023.109590 36858202 10.1016/j.radonc.2023.109590

[24] Karius A , Kreppner S , Strnad V , et al. Inter‐observer effects in needle reconstruction for temporary prostate brachytherapy: dosimetric implications and adaptive CBCT‐TRUS registration solutions. Brachytherapy. 2024;23(4):421‐432. doi:10.1016/j.brachy.2024.05.002 38845268 10.1016/j.brachy.2024.05.002

[25] Strnad V , Major T , Polgar C , et al. ESTRO‐ACROP guideline: interstitial multi‐catheter breast brachytherapy as Accelerated Partial Breast Irradiation alone or as boost—GEC‐ESTRO Breast Cancer Working Group practical recommendations. Radiother Oncol. 2018;128(3):411‐420. doi:10.1016/j.radonc.2018.04.009 29691075 10.1016/j.radonc.2018.04.009

